# Phosphorus-Doped Graphene Electrocatalysts for Oxygen Reduction Reaction

**DOI:** 10.3390/nano12071141

**Published:** 2022-03-29

**Authors:** Xinxing Zhan, Xin Tong, Manqi Gu, Juan Tian, Zijian Gao, Liying Ma, Yadian Xie, Zhangsen Chen, Hariprasad Ranganathan, Gaixia Zhang, Shuhui Sun

**Affiliations:** 1 School of Chemistry and Material Science, Guizhou Normal University, Guiyang 550001, China; zhanxinxing@gznu.edu.cn (X.Z.); gumanqi@gznu.edu.cn (M.G.); tianjuan@gznu.edu.cn (J.T.); 20010080258@gznu.edu.cn (Z.G.); maliying@gznu.edu.cn (L.M.); 2 Key Laboratory of Low-Dimensional Materials and Big data, Guizhou Minzu University, Guiyang 550025, China; xieyadian@gzmu.edu.cn; 3 Centre Énergie, Matériaux et Télécommunications, Institut National de la Recherche Scientifique (INRS), 1650 Boulevard Lionel-Boulet, Varennes, QC J3X 1P7, Canada; zhangsen.chen@inrs.ca (Z.C.); hariprasad.ranganathan@inrs.ca (H.R.); gaixia.zhang@inrs.ca (G.Z.)

**Keywords:** doped graphene, oxygen reduction reaction, phosphorus-doped, codoped

## Abstract

Developing cheap and earth-abundant electrocatalysts with high activity and stability for oxygen reduction reactions (ORRs) is highly desired for the commercial implementation of fuel cells and metal-air batteries. Tremendous efforts have been made on doped-graphene catalysts. However, the progress of phosphorus-doped graphene (P-graphene) for ORRs has rarely been summarized until now. This review focuses on the recent development of P-graphene-based materials, including the various synthesis methods, ORR performance, and ORR mechanism. The applications of single phosphorus atom-doped graphene, phosphorus, nitrogen-codoped graphene (P, N-graphene), as well as phosphorus, multi-atoms codoped graphene (P, X-graphene) as catalysts, supporting materials, and coating materials for ORR are discussed thoroughly. Additionally, the current issues and perspectives for the development of P-graphene materials are proposed.

## 1. Introduction

The energy conversion technique is one of the most important parts that significantly influence the development of human society. The conversion efficiency of converting chemical energy into electrical energy is many folds higher than that of conversion of chemical energy into thermal (e.g., combustion) or kinetic energies (e.g., modern IC engines). Accordingly, great attention has been given to the study of fuel cells and metal-air batteries as the next-generation energy conversion techniques because these devices can directly convert chemical energy into electric energy with nearly zero carbon footprint [1,2,3,4,5,6,7,8,9]. In fuel cells and metal-air batteries, the electrochemical reactions, in particular, the oxygen reduction reaction (ORR) in the cathode, determine the performance [10,11]. Hence, a suitable catalyst is highly needed to boost the kinetics and reduce the overpotential. Thus far, the noble metal platinum (Pt)-based materials have proven to be state-of-the-art catalysts for ORR [12,13]. However, the commercial application of Pt-based catalysts is hampered by their prohibitive cost, limited supply, and unsatisfactory durability. Therefore, developing cheap and earth-abundant alternatives with outstanding activity and excellent durability is of significance [14,15,16,17,18].

Graphene-based materials have attracted extensive interest in the field of electrocatalysis due to their fascinating mechanical, electronic, and thermal properties [19,20,21,22,23]. In particular, the high surface and the high conductivity are beneficial to the mass transfer and the electron transfer, thus increasing the activity of heterogeneous catalysis [17]. However, the pristine graphene has no intrinsic bandgap and shows poor electrocatalytic activity [24]. It is necessary to change the electronic density of the graphene sheet to modulate its electrochemical properties. When the carbon atoms in the graphene lattice are partially substituted by heteroatoms, the structure and electronic properties of the heteroatoms-doped graphene can be dramatically altered by the specific characters of the dopants. Usually, the neighboring elements of the carbon, such as nitrogen(N), boron(B), oxygen(O), phosphorus(P), sulfur(S), and fluorine(F), are preferred as the dopants for graphene [25,26]. Depending on their atom size, electronegativity, and doping level, the adsorption behavior, the chemisorption energy, and the active sites of the doped graphene can be modulated.

Among these elements, nitrogen is by far the most studied dopant because of its similar atomic size to carbon and slightly higher electronegativity than carbon. The ORR performance can be remarkably enhanced in nitrogen-doped graphene (N-graphene), which has been demonstrated and reviewed in previous works [27,28,29]. Besides, many previous articles reviewed the synthesis methods and the application of different heteroatoms-doped graphene (X-graphene) [25,30,31]. However, a limited number of articles focused on phosphorus-doped graphene (P-graphene). Therefore, to figure out the internal decisive factor of doped-graphene to affect the ORR performance, intensive research on P-graphene is indispensable.

**Figure 1:**
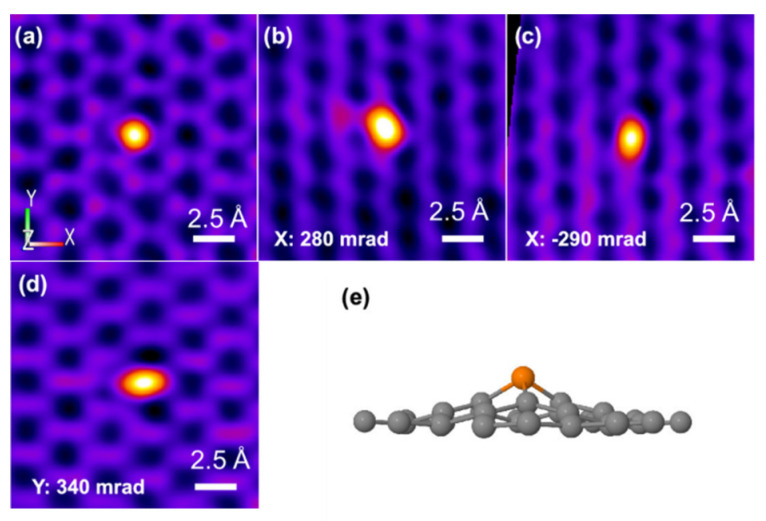
(**a**–**d**) Atomically resolved STEM of P-graphene with different angles along the X-axis and Y-axis (**e**) The experimental three-dimensional model of P-graphene. Reproduced with permission from [32]. Copyright © 2022, American Chemical Society.

**Figure 2:**
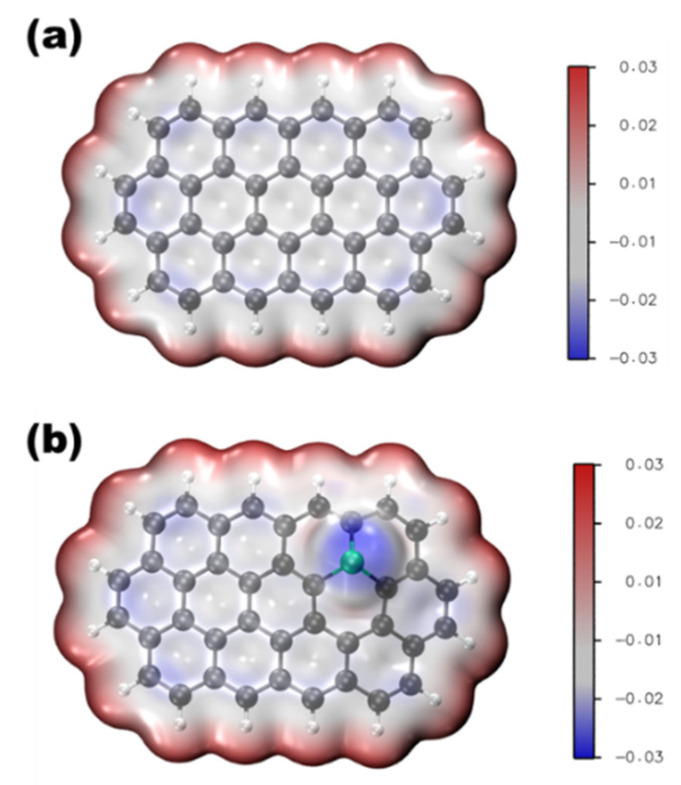
Electrostatic potential map of (**a**) pristine graphene, (**b**) P-graphene.

Phosphorus is located on the periodic table in Period 3 and Group 5. Like its congener, nitrogen, phosphorous has five electrons in the valence shell. The geometric and electronic effects of phosphorus doping differ from nitrogen doping because of its large atom size and low electronegativity compared to both nitrogen and carbon. As seen from the STEM images in Figure 1, there is a protrusion of the phosphorus atom above the plane of the graphene lattice in P-graphene because the atom size of phosphorus (98 pm) is larger than that of carbon (67 pm) [32]. The C–P bond and the nearest C–C bond are elongated, compared to the pristine C–C bond. Although nitrogen has the same valence number as phosphorus, the difference between P-graphene and N-graphene is distinct. In graphene, the pyramidal-like bonding phosphorus can lead to the transformation of sp^2^ hybridized carbon to sp^3^ state. These lattice distortion-induced defects can serve as the active site for ORR. Moreover, the phosphorus in P-graphene could act as a donor and show an n-type nature because of its weak attraction with the valence electrons in the M-shell. To further confirm the electronic structure difference between P-graphene and pristine graphene, the density functional theory (DFT) simulations are conducted. As in the electrostatic potential map in Figure 2a, the pristine graphene shows electroneutrality except for the unsaturated carbon atoms at the edge. In this case, the undoped graphene affords limited active sites and thus poor activity towards ORR. When the phosphorus is doped into graphene, as shown in Figure 2b, it could cause the redistribution of the surface charge. The carbon atoms near phosphorus are negatively charged. As the coordination environment changed, the adsorption behavior of the oxygen molecule and the binding strength of the intermediate species are changed [33]. Thus, it is interesting and worthwhile to carry out an in-depth study on how phosphorus impacts the catalytic performance toward ORR.

Accordingly, in this review, we discuss the recent studies of phosphorus-doped graphene, including phosphorus mono-doped (P-) and phosphorus with other elements codoped (P, X-) graphene. Herein, the works reported on phosphorus-doped graphene served as metal-free catalysts, supporting materials, and coating materials are reviewed. The synthesis methods, catalytic mechanism, and ORR performance are systematically summarized. The synergistic effect of inducing defects and electron distribution on the ORR activity and durability are also discussed. Finally, the current challenges and future perspectives in the field of P-graphene ORR catalysts are proposed.

## 2. The Synthesis of Phosphorus-Doped Graphene

**Figure 3:**
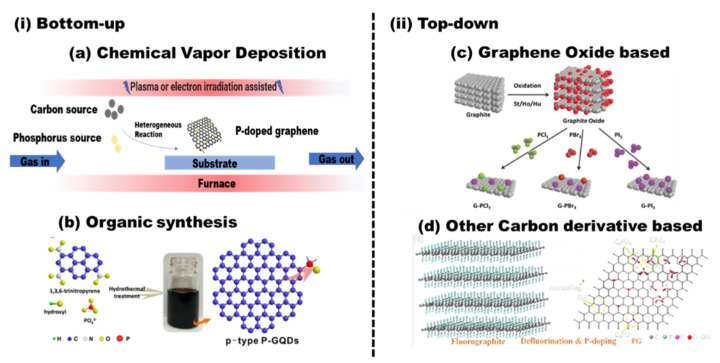
The synthesis routes of phosphorus-doped graphene: (**i**) bottom-up: (**a**) chemical vapor deposition (CVD) and (**b**) organic synthesis; reproduced with permission from [34] *©* 2022, American Chemical Society. (**ii**) Top-down: (**c**) graphene oxide-based method; reproduced with permission from [35] © 2022 Wiley-VCH VerlagGmbH & Co. KGaA, Weinheim; (**d**) other carbon derivative-based method; reproduced with permission from [36]; copyright © 2022, American Chemical Society.

It is well known that there are a large number of synthesis strategies to obtain graphene. According to the formation mechanism and the source of the carbon (small carbon molecule or graphite), the synthesis strategies can be divided into two types: top-down and bottom-up. The intrinsic properties and electronic structure of as-prepared doped graphene largely depend on the pristine graphene. Therefore, we divide these doping methods into two categories, as shown in Figure 3. In those strategies, if the heteroatoms partially replace some of the carbon atoms in the carbon skeleton simultaneously during the formation of graphene, it can be called in situ doping. If the doping is achieved based on the as-prepared graphene with dopant-containing precursors, it can be called post-treatment doping.

### 2.1. Bottom-up Approaches

The in situ doping is mainly based on the bottom-up pathway to produce graphene from small carbon molecules. Theoretically, the structure of the doped graphene can be fabricated on an atomic level. The doped graphene with high purity and fine structure can be obtained. However, this kind of method suffers from low yield, high cost, and difficulties in scaling up. Moreover, phosphorus is not easy to be introduced into the carbon skeleton because of its larger atom size than that of carbon. Engineering-desired, doped structures remain a challenge and detailed theoretical and experimental investigations are warranted.

#### 2.1.1. Chemical Vapor Deposition (CVD)

Chemical vapor deposition (CVD) is one of the most used technologies to produce low-dimensional materials, such as carbon nanotubes and graphene. It is based on the chemical reaction of the precursors in the vapor phase [37]. As shown in Figure 3a, the carbon source and phosphorus source in the gas phase are transported into the reactor to form thin P-graphene film on the heated substrate via homogeneous reactions. In L.G. Bulusheva’s work [38], P-graphene was deposited on copper substrates under low pressure of a gaseous mixture of CH_4_/PH_3_/H_2_, and the phosphorus content is about 0.1%. In the prepared P-graphene, the phosphorus atoms are located at the edge of graphene as phosphorus oxides, such as C–P=O or P–Ox. Triphenylphosphine (TPP) [39,40] can also be used as phosphorus sources. The nucleation, growth, and coalescence behavior of the P-graphene is largely determined by the temperature, the surface chemistry of the substrate, the pressure, and the precursor used.

After the graphene is grown on the catalyst surface, additional energy from external sources, including heating [41], ions implantation [42], electron irradiation [43], and laser direct writing [44], is essential to break the chemical bond to achieve doping. The phosphorus precursor can be triphenylphosphine [43], triphenylamine [41], P-ions [42], and phosphoric acid [44]. Depending on the doping method and the precursor used, a large range of P content can be obtained, 5.56% in [42], 4.96% in [41], 0.92% in [44], and 0.26% in [43] (based on XPS results). Interestingly, the phosphorus in those P-graphenes shows two kinds of bonding configurations, P–C and P–O [42,44].

Although high-quality doped graphene films can be obtained by CVD, complicated procedures, such as substrate pretreatment, or protection layer coating are needed. How to transfer the doped graphene from the catalyst surface onto arbitrary substrates without damaging the graphene is still a million-dollar question. Furthermore, the precise controlling of the P–C bond configuration is desired for further study.

#### 2.1.2. Organic Synthesis

The P-doping can also be achieved simultaneously during the formation of graphene by stepwise solution chemistry of organic precursors. As shown in Figure 3b, Na_2_HPO_4_ was used as a phosphorus source [34]. After hydrothermal treatment, the phosphorus in graphene mainly exists as CPO_3_/C_2_PO_2_ and C_3_PO structures. Similar methods were used and the tetrakis(hydroxymethyl)phosphonium chloride [45], Na_2_HPO_4_ [46], and sodium phytate [47] were used as phosphorus sources. Although this kind of method can effectively control the morphology and structures of the as-prepared products, the sizes of the P-graphenes are usually very small, called P-graphene dots. Their applications mainly focus on luminescence-related fields, such as photodegradation and photocatalyst.

### 2.2. Top-down Approaches

Top-down approaches mean the doped graphene comes from the graphite and graphite derivatives. To extract the graphene from graphite and graphite derivatives, different methods, such as sonication and hydrothermal, were used. The precursor mainly determines the structure and intrinsic properties of the as-prepared doped graphene. Graphite oxide (GO) is the most used precursor for doped graphene. Thus, we devote a separate chapter to this method. These methods are always cost-effective, easy to scale up, and have high yields. Yet, it is difficult to precisely control the configuration of the doped graphene.

#### 2.2.1. Graphite Oxide-Based Routine 

Graphite oxide (GO), which comes from the oxidation of layered graphite mainly by a modified hummers method, is a promising precursor and versatile building block for chemically converted graphene in large volumes owing to its tailorable surface chemistry [48]. After oxidation, the space between GO layers is increased, compared to that of the graphite, because of the repulsive force between hydrophilic oxygen-containing functional groups, such as peroxy, hydroxyl, epoxy, and carboxyl groups on the surface. The GO can be easily dispersed in water and conduct chemical modification further [49]. The larger number of oxygen functional groups and defeats on graphene can also play a critical role in heteroatoms doping. Therefore, the GO-based routine is by far the most used method to get heteroatoms-doped graphene, such as N-graphene, P-graphene, B-graphene, and P, N-graphene [50,51,52].

P-graphene can be obtained by simply mixing the phosphorus source with GO in solution [53]. The XPS results show that the leading P components are the P–O bond without reduction. To prepare the doped graphene with less oxygen content, the thermal treatment was conducted. Different chemicals, such as triphenylphosphine [54],1-butyl-3-methylimidazolium hexafluorophosphate [55], phosphoric acid [56], phytic acid [57], and tetrabutylphosphonium bromide [58], were used as phosphorus precursors. Usually, the mixture of GO and phosphorus precursors was annealed in high temperatures (more than 800 °C) under an inert atmosphere. In addition, a hydrothermal or solvothermal procedure can also be added to build three-dimensional (3D) P-graphene hydrogel [58,59,60,61]. After mixing the GO with phosphorus precursors, the solution was transferred to a Teflon-lined autoclave at high temperatures (180–220 °C); then, the annealing process was carried out. Additionally, the reduced GO with less residual oxygen functionalities was also used to prepare P-graphene [62].

Moreover, different combinations of phosphorus sources and heteroatom sources are used to prepare phosphorus and heteroatoms codoped graphene, such as P, F-graphene, P, N-graphene, and P, S-graphene [35,63,64,65,66,67,68]. As shown in Figure 3c, different phosphorus- and halogen-codoped graphene were obtained by refluxing the mixture containing phosphorus and halogen precursor. Three kinds of GO were synthesized by different methods [35]. The doping level of phosphorus and halogen depends on the type of GO. GO prepared by Hummers shows large amounts of dopant.

Because of the abundant oxygen-containing functional groups on GO, there is a considerable amount of oxygen content in the final P-graphene. Thus, there should be different P–O bonds and P–C bonds, such as C_3_–P=O, C–P–O, C–O–P. The presence of these phosphorus configurations plays a key role in electrochemical performance. We will discuss these impacts later in this article.

#### 2.2.2. Other Graphite Derivative-Based Routine

Graphite is thousands of graphene layers stacked together. The graphite was also used as the starting material to produce P-graphene by an electrochemical exfoliated method [69,70]. Because of the narrow space between graphite, the expanded graphite layer should be prepared by the intercalation of the solvent molecule. Then, the introduction of phosphorus can be achieved after the subsequent erosion/expansion process. Phosphoric acid (H_3_PO_4_) was used as a phosphorus source and electrolyte. Ball milling of graphene stack and red phosphorus were also used to produce P-graphene [71]. Generally, using graphite as the starting material, the phosphorus content in P-graphene can reach less than 1% (0.68% in [69], up to 0.7% in [70], 0.91% in [71]).

To enhance the phosphorus-doping content, fluorography was used [36]. As shown in Figure 3d, there are many vacancies in the graphene sheets, and the space between the graphene layer is large. Thus, it is easy to introduce phosphorus atoms, and the phosphorus content in P-graphene can reach 6.40% by thermal annealing in red phosphorus vapor.

## 3. Phosphorus-Doped Graphene for Metal-Free ORR Electrocatalyst

Metal-free catalyst based on carbon nanomaterials has been studied intensively as an emerging class of ORR catalysts since 2009 [72]. Recently, this kind of catalyst has been demonstrated to be a promising alternative ORR catalyst with low cost and high efficiency, as well as tolerance to methanol crossover and carbon-monoxide-poisoning effects. Although there is an argument that the metal-free catalyst strictly does not exists because of the residual metal on the catalyst, the active sites are mainly proven to be the adjacent area of the heteroatom. The doping-induced charge transfer in doped carbon material takes a crucial role to modulate the oxygen chemisorption mode and weaken the O–O bonding to facilitate the ORR [31,73,74]. Nitrogen or phosphorus single doping graphene was used in earlier research. Then, numerous works have been devoted to codoping graphene with a more complicated system.

### 3.1. Phosphorus-Doped Graphene

**Figure 4:**
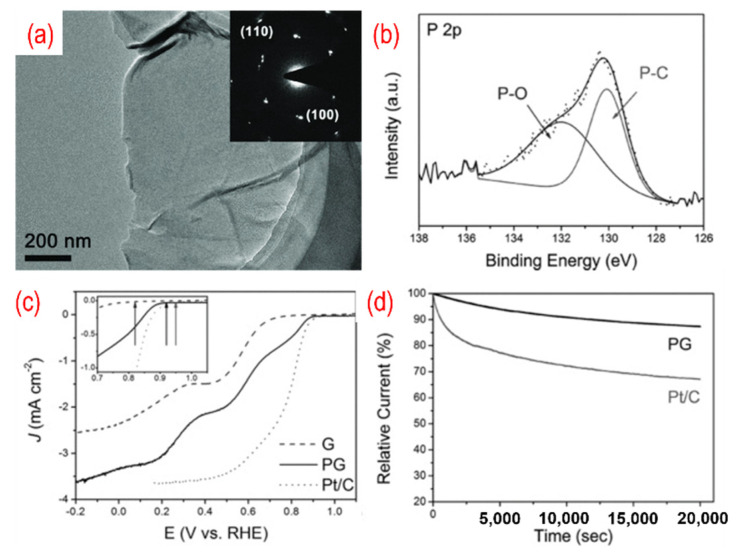
(**a**) The TEM image (the inset is the corresponding SAED pattern), (**b**) the high-resolution P 2p peaks, (**c**) the activity test (LSV curves recorded by RDE in O_2_-saturated 0.1 M KOH solution with 1600 rpm at a scanning rate of 10 mV s^−1^), (**d**) the stability test (current-time chronoamperometric in O_2_-saturated 0.1 M KOH solution) of P-graphene. Reproduced with permission from [54]. Copyright © 2022 WILEY-VCH Verlag GmbH & Co. KGaA, Weinheim.

The P-doped graphene made its debut as an ORR catalyst in 2013. Wei’s group simply annealed the mixture of GO and 1-butyl-3-methlyimidazolium hexafluorophosphate to achieve in situ P-doping of reduced graphene oxide (RGO) [55]. The phosphorus atoms were incorporated into the carbon frame with the formation of the C–P bond and the P–O bond, with the amount of phosphorus in P-graphene being 1.16%. The oxygen atoms are bonded with phosphorus atoms as C_3_PO, C_2_PO_2_, and CPO_3_. Thus, the polarized phosphorus serves as a bridge between the oxygen atoms and the carbon atoms. The carbon atom that bonded with a phosphorus atom and oxygen atoms is positively charged. Therefore, the active sites are these positively charged carbon atoms. P-graphene shows a one-step 4 e^−^ pathway and comparable ORR catalytic performance than that of the Pt/C catalyst. As shown in Figure 3, a similar method using GO and triphenylphosphine (TPP) as a precursor was used, and the phosphorus content is 1.81%. The TEM image in Figure 4a shows a typical crumpled surface. Besides, there are C–P bonds and the P–O bonds in P-graphene, as displayed in Figure 4b. The onset potential of P-graphene is 0.92 v vs. RHE and shows a combination of the 2 e^−^ and 4 e^−^ pathways (Figure 4c). Moreover, the prepared P-graphene (PG) maintains better stability than that of the Pt/C (Figure 4d). With the change of phosphorus source, similar methods thus show distinct results.

**Figure 5:**
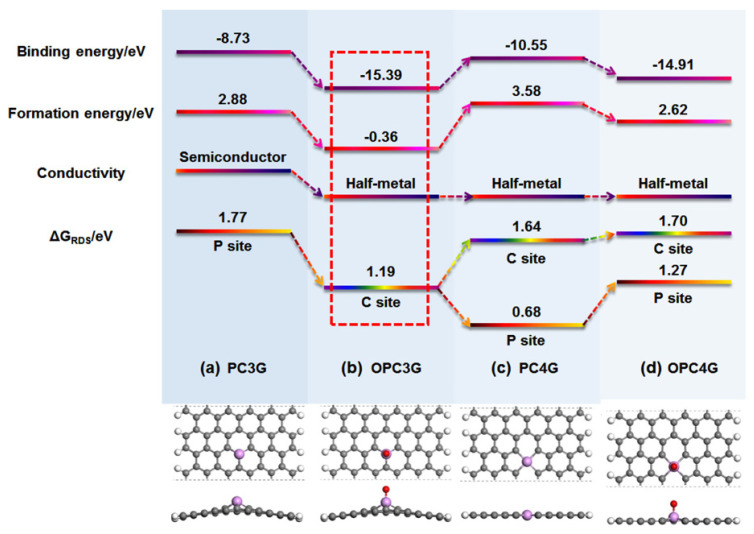
The simulation of different models of P-doped graphene for ORR performance. Reproduced with permission from [80]. Copyright © 2022, American Chemical Society.

Further theoretical and experimental works were conducted (some typical experimental works about P-graphene for ORR have been listed in Table 1) [75,76,77,78,79]. In Yang’s work, a phosphorus atom is believed to be the active site for ORR. The ORR could process by an indirect 2 e-pathway and the *OOH is intermediate [75]. Phosphorus-doped divacancy graphene was also used to study the reaction mechanism by Bai and co-workers. The ORR can take place on the phosphorus atom and its adjacent carbon atoms and demonstrates the 4 e- process [76]. As shown in Figure 5, four different models of P-doped graphene were considered to identify the relationships between chemical-bonding states and ORR performance by Wei’s group [80]. In their research, the impact of the oxygen, which can be doped into graphene lattice during the synthesis and electrochemical process were studied in which two possible structures, such as the OPC3G and PC4G, show the best ORR activity. The following simulation results show that it is difficult to directly form the PC4G structure while the OPC3G structure is more energetically favorable. The active sites for P-graphene could be the negatively charged carbon atoms in OPC3G. However, some scholars asserted that the positively charged phosphorus atoms can be regarded as active sites [25]. In summary, the ORR performance of P-doped graphene is attributed to many complicated factors, depending on the chemistry state of the precursor and synthesis method. It is still a long process to identifying the real mechanism of phosphorus-doped graphene for ORR.

**Table 1:** Summary of some typical works dedicated to P-graphene for ORR.

The Material ^1^	Synthesis Method	P-Content (at.%) ^2^	Onset Potential	Half-Wave Potential	Electron Transfer Number	Ref.
P-graphene	Annealing the mixture of GO and 1-butyl-3-methlyimidazolium	1.16%	−0.0261 V vs. SCE in 0.1 M KOH	~−0.2 V vs. SCE	3.9	[55]
P-graphene	Annealing the mixture of GO and triphenylphosphine	1.81%	0.92 V vs. RHE in 0.1 M KOH	-	3.0–3.8	[54]
P-graphene	Supercritical fluid processing of GO and triphenylphosphine	1.4–3.2% by EDX	0.12 V vs. MMO in 0.1 M KOH	-	-	[78]
P-graphene	Immersed into the mixture of GO and NaH_2_PO_4_	2.6%	~−0.3 V vs. Ag/AgCl in 0.1 M KOH	-	3.9	[79]
P, N-graphene	Pyrolysis of hexachlorocyclotriphosphazene (HCCP) and GO	1.08%	−0.20 V vs. Ag/AgCl	-	3.4–3.73	[81]
P, N-graphene	Twice pyrolysis treatment of GO and phosphoric acid	-	0.87 V vs. RHE in 0.1 M HClO4	0.64 V vs. RHE	-	[81]
P, N-graphene	Pyrolysis treatment of GO, polyaniline, and phytic acid	1.72%	1.01 V vs. RHE	~0.84 V vs. RHE	3.96	[82]
P, N-graphene	Two-step solution process using phytic acid and GO	0.6%	0.89 V vs. RHE	0.69 V vs. RHE	3.9	[83]
P, N-graphene	Pyrolysis treatment of GO and phytic acid	-	−0.11 V vs. Ag/AgCl	−0.34 V vs. Ag/AgCl	-	[84]
P, N-graphene	Hydrothermal and subsequent pyrolysis processes (GO and phytic acid)	1.22%	0.983 V vs. RHE	0.865 V vs. RHE	3.9–4.0	[85]
P, N-graphene	Pyrolysis treatment of phytic acid	0.67–0.71%	1.0 V vs. RHE	0.86 V vs. RHE		[86]
P, N-graphene	Pyrolysis treatment of GO and diammonium hydrogen phosphate	1.16%	~−0.2 V vs. Ag/AgCl	~−0.18 V vs. Ag/AgCl	3.66	[87]
P, N-graphene	Pyrolysis treatment of GO and diammonium phosphate	2.32%	~0.84 V vs. RHE	~0.87 V vs. RHE	3.99	[88]
P,Fe-graphene	Sol-gel polymerization and pyrolysis process	~2%	−0.139 V	-	3.74–3.89	[89]
P,Fe-graphene	Pyrolysis treatment of GO, phytic acid and FeCl_2_	0.84%	−0.05 V vs. Ag/AgCl in 0.5 M H_2_SO_4_	-	3.84	[90]
P,Co-graphene	Pyrolysis treatment of GO, tetraphenylphosphonium bromide, Co(NO_3_)_2_	0.639%	0.89 V vs. RHE	~0.78 V vs. RHE	3.87–3.96	[91]
N, P, F-graphene	Pyrolysis of the mixture of GO, polyaniline, and ammonium hexafluorophosphate	0.37%	~0.83 V vs. RHE	~0.72 V vs. RHE	3.85	[92]
P, S, N-graphene	Pyrolysis treatment of GO and acephate	0.42%	−0.192 V vs. SCE	-	2.99	[93]
P, S, N-graphene	Pyrolysis treatment of GO and phosphoric acid	-	−0.052 V vs. SCE	0.015 V vs. SCE	3.67–3.97	[94]
Fe, B, N, S, P-graphene	Pyrolysis treatment of GO and triphenylphosphine	0.54%	1.06 V vs. RHE	0.9 V vs. RHE	3.98	[95]
P, B, N-graphene	Hydrothermal method (GO and boron phosphate)	-	−0.12 V vs. Ag/AgCl	-	3.7	[96]
P, B, N-graphene	Pyrolysis treatment of GO and phenylphosphine	0.43%	0.88 V vs. RHE	0.80 V vs. RHE	3.8	[97]
P, Fe, N-graphene	Pyrolysis treatment of aphytic acid	1.11%	~0.95 V vs. RHE	0.84 V vs. RHE	3.2	[98]
P, Ni, N-graphene	Pyrolysis treatment of GO and phytic acid	-	0.88 V vs. RHE	-	3.5	[99]
P, S, N-graphene	Pyrolysis treatment of ammonium monohydrogenphosphate	0.95%	0.856 V vs. RHE	0.74 V vs. RHE	3.07	[100]
P, S, N-graphene	Ball milling and pyrolysis treatment of phosphonitrilic chloride trimer	1.16%	~0.93 V vs. RHE	0.88 V vs. RHE	-	[101]
MoP_x_ @MnP_y_ /P, N-graphene	Annealing the mixture of GO and the desired chemical	4.12%	0.965 V vs. RHE in 0.1 M KOH	0.842 V vs. RHE	3.95–3.97	[102]
CoMn_2_O_4_/ P, N-graphene	Hydrothermal method and soaking hypophosphorous acid	1.22%	−0.094 V vs. SCE in 0.1 M KOH	−0.2 V vs. SCE	3.64–3.70	[103]
Co/P, N-graphene	A hydrothermal method with the subsequent pyrolysis procedure	0.83% using elemental analysis	0.04 V vs. SCE	0.18 V vs. SCE	~4	[104]
Co_2_P /Co, P, N-graphene	Supramolecular gel-assisted strategy and annealing method	2.86%	0.90 V vs. RHE	0.81 V vs. RHE	3.96	[105]
Co_3_(PO_4_)_2_/P, N-graphene	Hydrothermal and annealing the mixture of desired chemical and phytic acid	-	0.95 V vs. RHE in 0.1 M KOH	0.81 V vs. RHE	3.7–3.84	[106]
Cu_3_P@ P, N-graphene	Annealing the mixture of desired chemical and 1-hydroxyethylidene1,1-diphosphonic acid	-	-	0.78 V vs. RHE	3.96–4.0	[107]
Co@N, P, S -graphene	Thermal treatment of the mixture of desired chemical and kelp	-	0.90 V vs. RHE	0.74 V vs RHE	4.0	[108]
FeCo@P, N-graphene/N-CNTs	Thermal treatment of the mixture of desired chemical and polystyrene spheres	2.77%	0.95 V vs. RHE	-	3.67–3.82	[109]
FeP@P-graphene	Annealing of the mixture of hemin diammonium phosphate and melamine	1.1%	0.95 V vs. RHE in 0.1 M KOH	0.81 V vs. RHE	3.8	[110]

Notes: ^1^ X/Y means Y-supported X. For instance, Co/P, N-graphene means P, N-doped graphene-supported Co. X@Y means X coating with Y. For instance, Co@N, P, S -graphene means Co coating with N, P, S-graphene. ^2^ The content is based on the XPS results unless the exception is in the chart.

### 3.2. P, N Codoped Graphene

**Figure 6:**
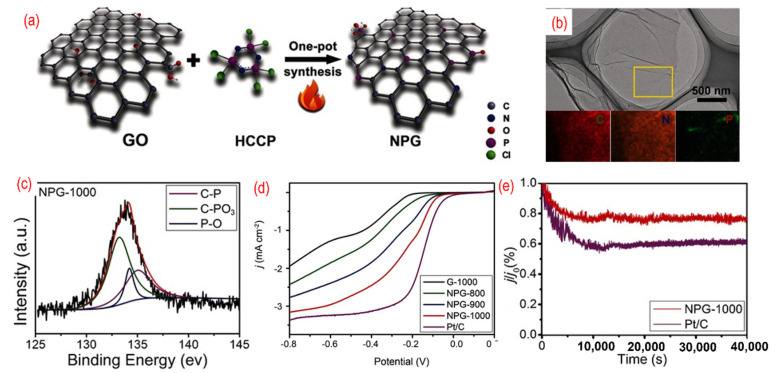
(**a**) The schematic of the synthesis process, (**b**) the TEM image (the inset is C-, N-, and P-elemental mappings), (**c**) the high-resolution P 2p peaks, (**d**) the activity test (LSV curves recorded by RDE in O_2_-saturated 0.1 M KOH solution with 1600 rpm at a scanning rate of 10 mV s^−1^), (**e**) the stability test (current-time chronoamperometric in O_2_-saturated 0.1 M KOH solution) of P, N-graphene. Reproduced with permission from [81]. © 2022 WILEY-VCH Verlag GmbH & Co. KGaA, Weinheim.

Codoping of the heteroatoms, such as nitrogen and boron, has been proven to improve the ORR performance. Among them, P, N-graphene has become a hotspot of research and shows superior electrocatalysts performances. As shown in Figure 6, L. Dong et al. reported on N, P- graphene that was prepared by the pyrolysis of hexachlorocyclotriphosphazene (HCCP) and GO [75]. Because the P–Cl in N_3_P_3_Cl_6_ with exatomic ring structure is prone to break under high temperature, the residual active groups containing N and P atoms can be incorporated into the graphene. The morphology of P, N-graphene is similar to pristine graphene and the N and P atoms are homogeneously distributed on the graphene sheets, as shown in Figure 6b. In the XPS spectra of the as-obtained P, N-graphene, three chemical bonds, such as C–P, C–PO_3_, and P–O, are found. The formation of P–O is related to the active oxygen released from GO under high temperatures. The content of C–P increases with the increase of the annealing temperature. The annealing temperature was adjusted to estimate the effect on ORR. The onset potential of optimized P, N-graphene (−0.20 V vs./AgCl) annealed under 1000 °C is close to that of the commercial Pt/C (−0.02 V vs./AgCl) and shows better electrochemical stability (Figure 6c,d). The best catalyst with the highest annealing temperature can result in a high degree of graphitization and excellent conductivity. Moreover, the synergistic effects of N, P-codoping and the suitable ratio of nitrogen and phosphorus can produce the asymmetrical spin and charge density, thus enhancing the catalytic activity. In an acidic environment, the P, N-graphene shows moderate ORR performance [111]. The onset potential of the P, N-graphene is 0.87 v vs. RHE, and the limiting current is only around 3.5 mA cm^−2^. Lately, phytic acid [82,83,84,85,86], ammonium hydrogen phosphate [87], and diammonium phosphate [88] were also used as the phosphorus source to obtain N, P-graphene.

**Figure 7:**
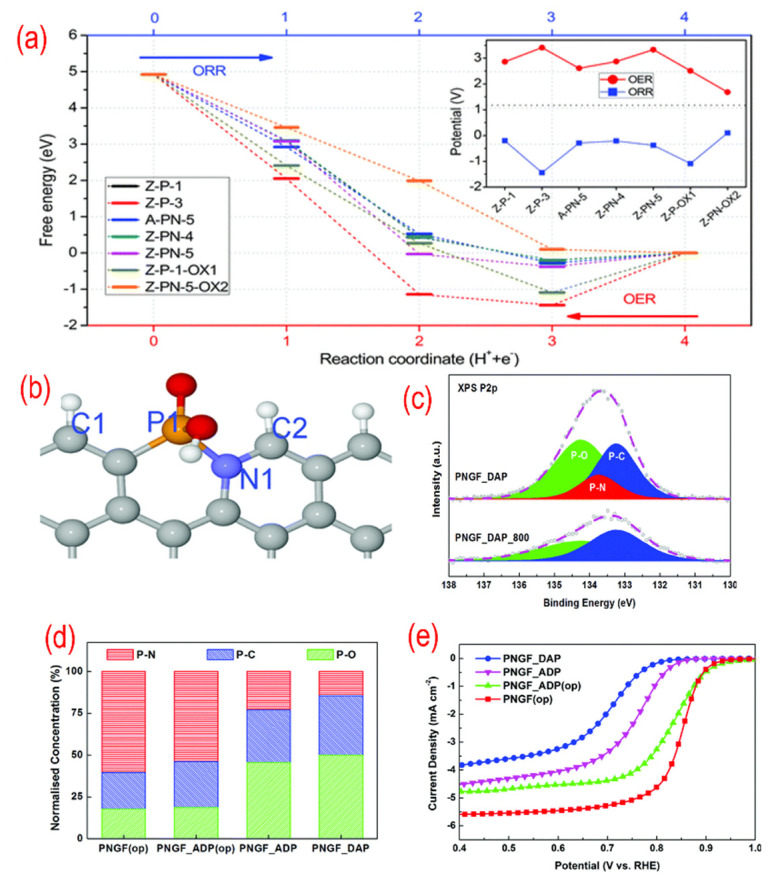
(**a**) The free energy variations of different P-containing structures for each ORR step. (**b**)The favorable N, P-containing structures. (**c**) The high-resolution P 2p peaks of N, P-graphene. (**d**) The relative ratio of XPS P2p binding configurations for different N, P-graphene materials. (**e**) The activity test (LSV curves recorded by RDE in O_2_-saturated 0.1 M KOH solution with 1600 rpm at a scanning rate of 10 mV s^−1^) for different N, P-graphene materials. Reproduced from [88] with permission from the Royal Society of Chemistry.

To clarify the real active sites in P, N-graphene, the theoretical works were conducted [88,112,113]. As shown in Figure 7, the free energy of different P-containing structures for ORR are calculated by DFT simulation. The active sites of P, N- graphene are the edge carbon atoms next to the N–P bond as C2 in Figure 7b. To get the high concentration of P–N bonds, the dosage of the precursor was adjusted according to the P/N ratio. The optimal P, N-graphene with the highest P–N bond and lowest NH_2_ group concentration shows the best ORR performance.

### 3.3. Phosphorus, X Codoped Graphene

Codoping other elements except nitrogen with phosphorus have been studied to figure out the relationship between the redistributed electronic state of graphene and the catalytical performance. Phosphorus and metal, such as Co, Fe codoped catalysts, were developed as ORR catalysts [89,90,114]. The metal P bond, such as Fe–Px or Co-P, can always be found and serve as active sites.

**Figure 8:**
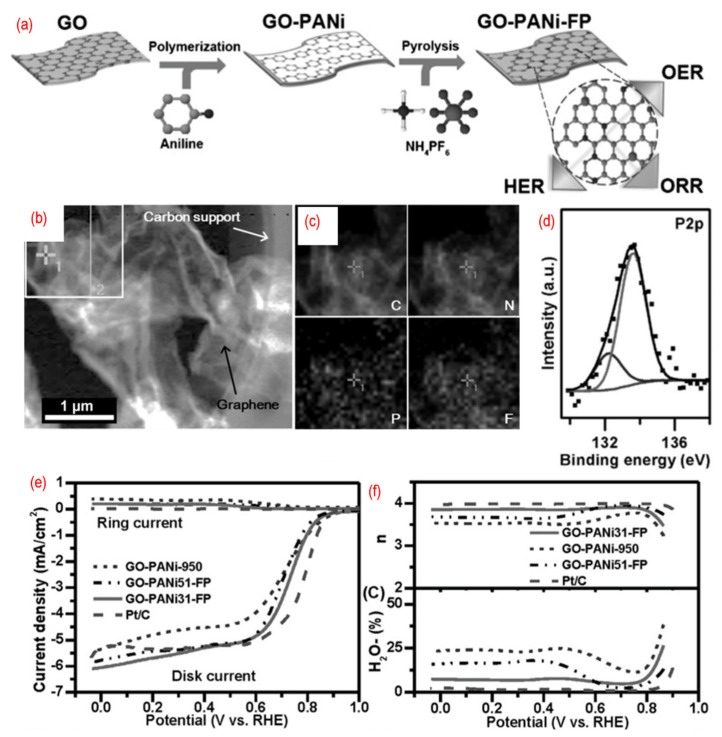
(**a**) The schematic of the synthesis process, (**b**) the TEM image, (**c**) C-, N-, F-, and P-elemental mappings, (**d**) the high-resolution P 2p peaks, (**e**) the activity test (LSV curves recorded by RRDE in O_2_-saturated 0.1 M KOH solution with 1600 rpm at a scanning rate of 10 mV s^−1^), (**f**) the electrons transfer number and H_2_O_2_ yield of N, P, F-graphene. Reproduced from [92]. © 2022 WILEY-VCH Verlag GmbH & Co. KGaA, Weinheim.

Furthermore, the third element can also be introduced into graphene lattice to get tridoped graphene. As shown in Figure 8, N-, P-, and F-tridoped graphenes are synthesized by pyrolysis of the mixture of GO, polyaniline, and ammonium hexafluorophosphate. The phosphorus, nitrogen, and fluorine are homogeneously doped into wrinkled graphene, thus introducing defects and changing the surface properties. There are P–C bonds and P–O bonds in N, P, F-graphene. The third element, fluorine, can induce strong charge redistribution for the carbon atom in the graphene. The N, P, F-graphene show a 4 e- pathway for ORR. The P, S, N-graphene [93,94,100,101], P, N, Fe-graphene [91,98], Fe, B, N, S, P-graphene [95], P, N, B-graphene [96,97], and Ni, N, P-graphene [99] are developed as well. Significantly, these materials always served as the multifunctional catalyst for ORR, OER, HER, and carbon dioxide reduction reaction (CO_2_RR). This is mainly related to the different charged sites of the graphene due to the different heteroatoms doping.

## 4. Phosphorus-Doped Graphene Composite for ORR

### 4.1. P-Graphene as Catalyst Support Material for ORR

The nanosized elemental metal, metal oxide, metal nitrides, and metal phosphides have potentially served as ORR catalysts. However, these nanoparticles are prone to agglomerate and aggregate during the synthesis and operation process because of the high surface energy. Besides, these nanosized catalysts always suffer from limited active surface areas and poor charge conductivity. Therefore, the substrate with a high surface area is generally used to disperse the nanoparticles. Doped graphene is a good candidate to accelerate the homogeneous dispersion. In particular, phosphorus atoms with multielectron orbital in P-graphene can lead to enhanced interactions between the catalyst and supports. The surface chemical state of P-graphene can not only affect the morphology but also modulate the electronic structure of the catalysts. A few research works have been devoted to using the P-graphene as catalyst support for ORR [102,103,104,105,106].

**Figure 9:**
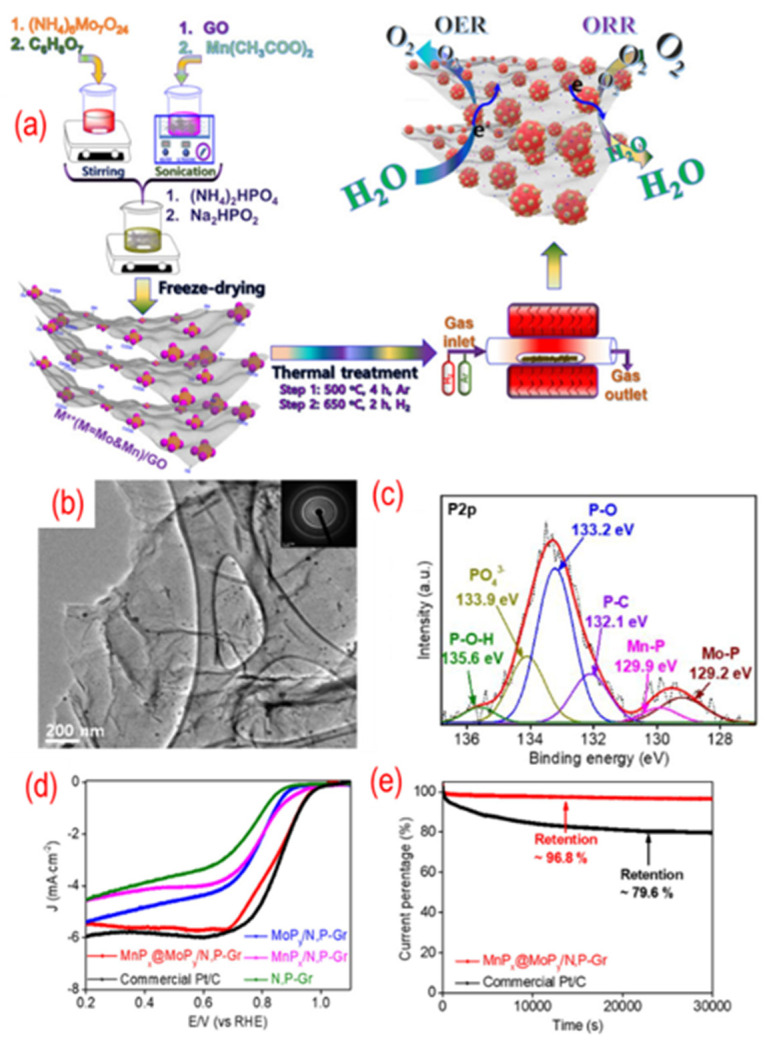
(**a**) The schematic of the synthesis process, (**b**) the TEM image (the inset is the corresponding SAED pattern), (**c**) the high-resolution P 2p peaks, (**d**) the activity test (LSV curves recorded by RDE in O2-saturated 0.1 M KOH solution with 1600 rpm at a scanning rate of 10 mV s^−1^), (**e**) the stability test (current-time chronoamperometric in O2-saturated 0.1 M KOH solution) of MnPx@MoPy supported on P, N-graphene. Reproduced with permission from [102]. Copyright © 2022, American Chemical Society.

As shown in Figure 9, MoP_x_@MnP_y_ nanoparticles on P, N-graphene were obtained by annealing the mixture of GO and the desired chemicals. The phosphorus is (NH_4_)_2_HPO_4_ and Na_2_HPO_2_·H_2_O. The MoP_x_@MnP_y_ homogeneously disperse on the P, N-graphene due to the negatively charged state of nitrogen and phosphorus codoping. There are P–C, Mn–P, Mo–P, P–O, P–O–H, and PO_4_ in the hybrid structure, indicating the enhanced interaction between MoP_x_@MnP_y_ and P, N-graphene. Hence, the composite shows good electrocatalytic activity and stability. Besides, different CoMn_2_O_4_, cobalt, Co_2_P, and cobalt phosphate nanoparticles are also dispersed on the P, N-graphene [103,104,105,106]. These works are also summarized in Table 1.

### 4.2. P-Graphene as a Coating Material for ORR

Encapsulating the active nanosized catalyst into carbon materials is another way to resist aggregation and agglomeration by dual chemical and physical protection. Furthermore, the structural and electronic properties of the active catalyst can be modulated with the contact of the doped carbon materials. The carbon materials outside the catalyst can be called graphene or carbon shells in different literature. When the thickness of this carbon layer is very thin, it is called graphene in this review.

**Figure 10:**
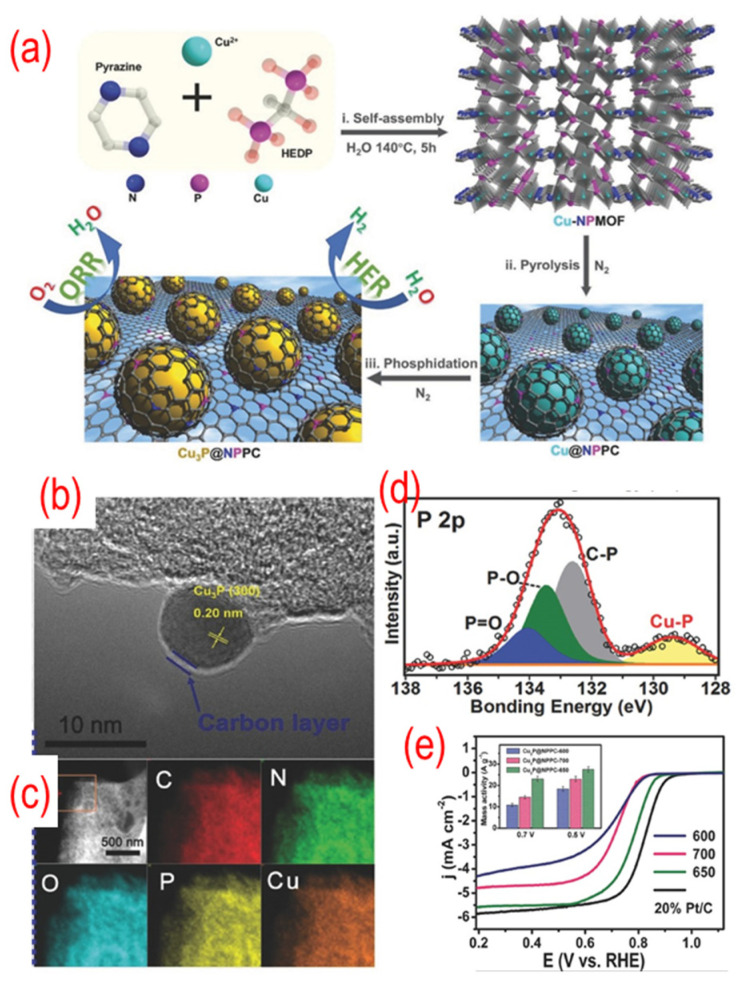
(**a**) The schematic of the synthesis process, (**b**) the TEM image, (**c**) C-, N-, O-, P-, and Cu-elemental mappings, (**d**) the high-resolution P 2p peaks, (**e**) the activity test (LSV curves recorded by RDE in O_2_-saturated 0.1 M KOH solution with 1600 rpm at a scanning rate of 10 mV s^−1^), of Cu_3_P@ P, N-graphene. The inset of (**e**) is the mass activity of Cu_3_P@ P, N-graphene catalysts. Reproduced with permission from [107]. © 2022 Wiley-VCH Verlag GmbH & Co. KGaA, Weinheim.

As shown in Figure 10, the precursors of the Cu_3_P, which were obtained by self-assembly, and 1-hydroxyethylidene-1,1-diphosphonic acid (HEDP) were annealed under N_2_ atmosphere to obtain Cu_3_P@P, N-graphene. The TEM image shows that the Cu_3_P NPs are covered with a thin carbon shell. The N and P atoms in the precursor play a vital role in incorporating Cu_3_P NPs into the carbon network and in situ doping nitrogen and phosphorus. A strong interaction can be found between the Cu_3_P and P, N-graphene because of the chemical bonding of copper with phosphorus. The optimized Cu_3_P@P, N-graphene shows comparable catalytic activity to that of the Pt/C catalyst because of the synergistic effect between the Cu_3_P and P, N-graphene shell. Later, similar strategies were used by different groups to prepare Co@N, P, S -graphene, FeCo@P, N-graphene/N-CNTs, and FeP@P-graphene [108,109,110]. Interestingly, these materials are always used as bifunctional catalysts, such as OER, HER, and triiodide reduction reaction (TIRR). As a result, presently, this type of P-graphene encapsulating technology is gaining particular attention.

## 5. Perspectives

Regardless of abundant works done on the P-graphene, continuous efforts are needed to rationally design and fabricate the highly efficient ORR catalysts. Some current challenges and future perspectives are listed below.

First, the precisely controlled synthesis for heteroatom-doped graphene remains a key challenge and needs to be explored further. In present catalysts, there are many types of dopants and various defects. The electrochemical reaction is delicate, so it is difficult to identify the real factors that are responsible for enhancing the ORR activity. The synergistic mechanism is one of the most used explanations for the improvement of ORR. However, it is essential to clarify the effect of a specific chemical bond. Doping desired heteroatom(s) with a dominated specific type of chemical bond in a particular position is the first step to studying the doping effect. Currently, GO is being used as the carbon support in most of the research related to electrocatalysis. The conventional pyrolysis of GO cannot meet the demands. The changing structure and surface chemistry of GO during thermal treatment should be tuned. In addition, the controllable synthetic routes should be further developed. For example, magnetron sputtering is one of the promising physical vapor deposition techniques for the preparation of metal, alloy, and compound thin films. Under a high vacuum environment with a magnetically confined plasma, the atoms from the target can be sputtered onto the substrates. P, S, F, and N are commonly used as dopants by this technique [115,116,117]. Furthermore, the catalyst layer can be directly deposited on the membrane of the membrane electrode assembly (MEA) in fuel cells by magnetron sputtering.

Secondly, more efforts should be made on the physical property evaluations of P-graphene, such as thermal stability, wettability, and thermal conductivity, which also play important roles in ORR performance. It is also important for the synthesis routes. For example, the defects can be found in sing layer-graphene prepared by CVD at ~500 °C, while defects appear in bilayer graphene fabricated at ~600 °C [118]. More studies should be done on the physical property characterizations to achieve controlled production of P-graphene.

Thirdly, identifying the types and quantifying the content of dopants accurately is still a big challenge. XPS is a frequently used technology to study the information of chemical bonding. However, even with a similar catalyst, the analysis of XPS data varies between different research groups. Other advanced characterization techniques, such as X-ray absorption spectroscopy, should be involved.

Fourthly, the underlying catalytic mechanism, as well as the real active sites for ORR of doped graphene is not yet identified clearly. Although some theoretical simulations have been done, there is still a big gap between the theoretical model and the actual working state of the catalysts. Some well-designed experiments and in situ characterizations should be done to unveil the catalytic mechanism.

Last but not least, the building of the multiple doped structures or the composites will play a more important role. For example, we have recently synthesized P, N-graphene dots/N-3D-graphene [27]. We found that P–N bonded structures can serve as ORR active sites. Because of the edge defects and abundant functional groups, the catalyst shows excellent ORR performance. Also, the P-doped graphene can be used as the support for metal catalysts.

## 6. Conclusions

In summary, doping graphene with phosphorus and various heteroatoms has been proven as a promising way to enhance the ORR performance. More importantly, the experimental and theoretical studies of P-graphene are essential to study the catalytic mechanisms towards ORR, thus providing a general strategy to obtain the advanced non-noble metal catalysts for the application of energy conversion devices, such as fuel cells and metal-air batteries. In this review, the recent advances in P-doped graphene with an overview of the various synthesis methods, ORR performance, and ORR mechanism are discussed. Furthermore, we believe that these achievements can be used in other energy conversion and storage fields. Further efforts on the study of P-graphene are warranted.

## Data Availability

All raw data in this study can be provided by the corresponding authors on request.

## References

[1] Mu Y., Wang T., Zhang J., Meng C., Zhang Y., Kou Z. (2022). Single-atom catalysts: Advances and challenges in metal-support interactions for enhanced electrocatalysis. Electrochem. Energy Rev..

[2] Ren X.F., Liu B.H., Liang X.Y., Wang Y.R., Lv Q.Y., Liu A.M. (2021). Review-current progress of non-precious metal for ORR based electrocatalysts used for fuel cells. J. Electrochem. Soc..

[3] Zhao Z.H., Li M.T., Zhang L.P., Dai L.M., Xia Z.H. (2015). Design principles for heteroatom-doped carbon nanomaterials as highly efficient catalysts for fuel cells and metal-air batteries. Adv. Mater..

[4] Zhang J.T., Zhao Z.H., Xia Z.H., Dai L.M. (2015). A metal-free bifunctional electrocatalyst for oxygen reduction and oxygen evolution reactions. Nat. Nanotechnol..

[5] Liu J., Song P., Ning Z.G., Xu W.L. (2015). Recent advances in heteroatom-doped metal-free electrocatalysts for highly efficient oxygen reduction reaction. Electrocatalysis.

[6] Bai L., Zhang Y., Tong W., Sun L., Huang H., An Q., Tian N., Chu P.K. (2020). Graphene for energy storage and conversion: Synthesis and interdisciplinary applications. Electrochem. Energy Rev..

[7] Han A., Zhang Z., Yang J., Wang D., Li Y. (2021). Carbon-supported single-atom catalysts for formic acid oxidation and oxygen reduction reactions. Small.

[8] Xu X., Sun H., Jiang S.P., Shao Z. (2021). Modulating metal–organic frameworks for catalyzing acidic oxygen evolution for proton exchange membrane water electrolysis. SusMat.

[9] Xu Z., Deng W., Wang X. (2021). 3D hierarchical carbon-rich micro-/nanomaterials for energy storage and catalysis. Electrochem. Energy Rev..

[10] Tang L., Xu Q., Zhang Y., Chen W., Wu M. (2022). MOF/PCP-based electrocatalysts for the oxygen reduction reaction. Electrochem. Energy Rev..

[11] Cui X., Luo Y., Zhou Y., Dong W., Chen W. (2021). Application of functionalized graphene in Li–O2 batteries. Nanotechnology.

[12] Zhu S., Wang X., Luo E., Yang L., Chu Y., Gao L., Jin Z., Liu C., Ge J., Xing W. (2020). Stabilized Pt cluster-based catalysts used as low-loading cathode in proton-exchange membrane fuel cells. ACS Energy Lett..

[13] Tong X., Wei Q., Zhan X., Zhang G., Sun S. (2017). The new graphene family materials: Synthesis and applications in oxygen reduction reaction. Catalysts.

[14] Khan K., Tareen A.K., Aslam M., Zhang Y., Wang R., Ouyang Z., Gou Z., Zhang H. (2019). Recent advances in two-dimensional materials and their nanocomposites in sustainable energy conversion applications. Nanoscale.

[15] Wang Y., Li J., Wei Z. (2018). Recent progress of carbon-based materials in oxygen reduction reaction catalysis. Chemelectrochem.

[16] Ji Y., Dong H., Liu C., Li Y. (2018). The progress of metal-free catalysts for the oxygen reduction reaction based on theoretical simulations. J. Mater. Chem. A.

[17] Tong X., Zhan X., Rawach D., Chen Z., Zhang G., Sun S. (2020). Low-dimensional catalysts for oxygen reduction reaction. Prog. Nat. Sci. Mater. Int..

[18] Zhao S., Wang D.W., Amal R., Dai L. (2019). Carbon-based metal-free catalysts for key reactions involved in energy conversion and storage. Adv. Mater..

[19] Choi H.J., Jung S.M., Seo J.M., Chang D.W., Dai L.M., Baek J.B. (2012). Graphene for energy conversion and storage in fuel cells and supercapacitors. Nano Energy.

[20] Zhang J., Zhao F., Zhang Z., Chen N., Qu L. (2013). Dimension-tailored functional graphene structures for energy conversion and storage. Nanoscale.

[21] Wang D.-W., Su D. (2014). Heterogeneous nanocarbon materials for oxygen reduction reaction. Energy Environ. Sci..

[22] Geng D.S., Ding N., Hor T.S.A., Liu Z.L., Sun X.L., Zong Y. (2015). Potential of metal-free "graphene alloy" as electrocatalysts for oxygen reduction reaction. J. Mater. Chem. A.

[23] Jiang Y., Guo F., Liu Y., Xu Z., Gao C. (2021). Three-dimensional printing of graphene-based materials for energy storage and conversion. SusMat.

[24] Mazanek V., Luxa J., Matejkova S., Kucera J., Sedmidubsky D., Pumera M., Sofer Z. (2019). Ultrapure graphene is a poor electrocatalyst: Definitive proof of the key role of metallic impurities in graphene-based electrocatalysis. ACS Nano.

[25] Wang B., Liu B., Dai L. (2021). Non-N-doped carbons as metal-free electrocatalysts. Adv. Sustain. Syst..

[26] Kumar R., Sahoo S., Joanni E., Singh R.K., Maegawa K., Tan W.K., Kawamura G., Kar K.K., Matsuda A. (2020). Heteroatom doped graphene engineering for energy storage and conversion. Mater. Today.

[27] Tong X., Cherif M., Zhang G.X., Zhan X.X., Ma J.G., Almesrati A., Vidal F., Song Y.J., Claverie J.P., Sun S.H. (2021). N, P-codoped graphene dots supported on N-doped 3D graphene as metal-free catalysts for oxygen reduction. ACS Appl. Mater. Inter..

[28] Xue Y., Wu B., Bao Q., Liu Y. (2014). Controllable synthesis of doped graphene and its applications. Small.

[29] Wood K.N., O’Hayre R., Pylypenko S. (2014). Recent progress on nitrogen/carbon structures designed for use in energy and sustainability applications. Energy Environ. Sci..

[30] Poh H.L., Pumera M. (2015). P-element-doped graphene: Heteroatoms for electrochemical enhancement. Chemelectrochem.

[31] Dai L., Xue Y., Qu L., Choi H.J., Baek J.B. (2015). Metal-free catalysts for oxygen reduction reaction. Chem. Rev..

[32] Langer R., Błoński P., Hofer C., Lazar P., Mustonen K., Meyer J.C., Susi T., Otyepka M. (2020). Tailoring electronic and magnetic properties of graphene by phosphorus doping. ACS Appl. Mater. Inter..

[33] Yang N., Li L., Li J., Ding W., Wei Z. (2018). Modulating the oxygen reduction activity of heteroatom-doped carbon catalysts via the triple effect: Charge, spin density and ligand effect. Chem. Sci..

[34] Qian J., Shen C., Yan J., Xi F., Dong X., Liu J. (2018). Tailoring the electronic properties of graphene quantum dots by P doping and their enhanced performance in metal-free composite photocatalyst. J. Phys. Chem. C.

[35] Wang L., Sofer Z., Zboril R., Cepe K., Pumera M. (2016). Phosphorus and halogen co-doped graphene materials and their electrochemistry. Chem.-Eur. J.

[36] Lin L.H., Fu L., Zhang K.Y., Chen J., Zhang W.L., Tang S.L., Du Y.W., Tang N.J. (2019). P-superdoped graphene: Synthesis and magnetic properties. ACS Appl. Mater. Inter..

[37] Sun L., Yuan G., Gao L., Yang J., Chhowalla M., Gharahcheshmeh M.H., Gleason K.K., Choi Y.S., Hong B.H., Liu Z. (2021). Chemical vapour deposition. Nat. Rev. Methods Primers.

[38] Bulusheva L.G., Arkhipov V.E., Popov K.M., Sysoev V.I., Makarova A.A., Okotrub A.V. (2020). Electronic structure of nitrogen- and phosphorus-doped graphenes grown by chemical vapor deposition method. Materials.

[39] Ovezmyradov M., Magedov I.V., Frolova L.V., Chandler G., Garcia J., Bethke D., Shaner E.A., Kalugin N.G. (2015). Chemical vapor deposition of phosphorous- and boron-doped graphene using phenyl-containing molecules. J. Nanosci. Nanotechnol..

[40] Larrude D.G., Garcia-Basabe Y., Freire Junior F.L., Rocco M.L.M. (2015). Electronic structure and ultrafast charge transfer dynamics of phosphorous doped graphene layers on a copper substrate: A combined spectroscopic study. RSC Adv..

[41] Some S., Kim J., Lee K., Kulkarni A., Yoon Y., Lee S., Kim T., Lee H. (2012). Highly air-stable phosphorus-doped n-type graphene field-effect transistors. Adv. Mater..

[42] He S.-M., Huang C.-C., Liou J.-W., Woon W.-Y., Su C.-Y. (2019). Spectroscopic and electrical characterizations of low-damage phosphorous-doped graphene via ion implantation. ACS Appl.Mater. Inter..

[43] Shin D.W., Kim T.S., Yoo J.B. (2016). Phosphorus doped graphene by inductively coupled plasma and triphenylphosphine treatments. Mater. Res. Bull..

[44] Rao Y.F., Yuan M., Luo F., Wang Z.P., Li H., Yu J.B., Chen X.P. (2021). One-step laser fabrication of phosphorus-doped porous graphene electrodes for high-performance flexible microsupercapacitor. Carbon.

[45] Liu R., Zhao J., Huang Z., Zhang L., Zou M., Shi B., Zhao S. (2017). Nitrogen and phosphorus co-doped graphene quantum dots as a nano-sensor for highly sensitive and selective imaging detection of nitrite in live cell. Sens. Actuators B Chem..

[46] Guo Z., Wu H., Li M., Tang T., Wen J., Li X. (2020). Phosphorus-doped graphene quantum dots loaded on TiO_2_ for enhanced photodegradation. Appl. Surf. Sci..

[47] Wang W.J., Xu S.F., Li N., Huang Z.Y., Su B.Y., Chen X.M. (2019). Sulfur and phosphorus co-doped graphene quantum dots for fluorescent monitoring of nitrite in pickles. Spectrochim Acta A.

[48] Compton O.C., Nguyen S.T. (2010). Graphene oxide, highly reduced graphene oxide, and graphene: Versatile building blocks for carbon-based materials. Small.

[49] Gao W., Alemany L.B., Ci L., Ajayan P.M. (2009). New insights into the structure and reduction of graphite oxide. Nat. Chem..

[50] Li J.-C., Hou P.-X., Liu C. (2017). Heteroatom-doped carbon nanotube and graphene-based electrocatalysts for oxygen reduction reaction. Small.

[51] Feng L., Qin Z., Huang Y., Peng K., Wang F., Yan Y., Chen Y. (2020). Boron-, sulfur-, and phosphorus-doped graphene for environmental applications. Sci. Total Environ..

[52] Kaushal S., Kaur M., Kaur N., Kumari V., Singh P.P. (2020). Heteroatom-doped graphene as sensing materials: A mini review. RSC Adv..

[53] Some S., Shackery I., Kim S.J., Jun S.C. (2015). Phosphorus-doped graphene oxide layer as a highly efficient flame retardant. Chem.-Eur. J..

[54] Zhang C., Mahmood N., Yin H., Liu F., Hou Y. (2013). Synthesis of phosphorus-doped graphene and its multifunctional applications for oxygen reduction reaction and lithium ion batteries. Adv. Mater..

[55] Li R., Wei Z., Gou X., Xu W. (2013). Phosphorus-doped graphene nanosheets as efficient metal-free oxygen reduction electrocatalysts. RSC Adv..

[56] An M.C., Du C.Y., Du L., Sun Y.R., Wang Y.J., Chen C., Han G.K., Yin G.P., Gao Y.Z. (2017). Phosphorus-doped graphene support to enhance electrocatalysis of methanol oxidation reaction on platinum nanoparticles. Chem. Phys. Lett..

[57] Chu K., Wang F., Tian Y., Wei Z. (2017). Phosphorus doped and defects engineered graphene for improved electrochemical sensing: Synergistic effect of dopants and defects. Electrochim. Acta.

[58] Tian Y., Wei Z., Zhang K.H., Peng S., Zhang X., Liu W.M., Chu K. (2017). Three-dimensional phosphorus-doped graphene as an efficient metal-free electrocatalyst for electrochemical sensing. Sens. Actuators B-Chem..

[59] Zhang X., Wang K.P., Zhang L.N., Zhang Y.C., Shen L. (2018). Phosphorus-doped graphene-based electrochemical sensor for sensitive detection of acetaminophen. Anal. Chim. Acta.

[60] Fan X., Xu H., Zuo S.S., Liang Z.P., Yang S.H., Chen Y. (2020). Preparation and supercapacitive properties of phosphorus-doped reduced graphene oxide hydrogel. Electrochim. Acta.

[61] Nie G.S., Deng H.C., Huang J., Wang C.Y. (2020). Phytic acid assisted formation of phosphorus-doped graphene aerogel as electrode material for high-performance supercapacitor. Int. J. Electrochem. Sci..

[62] Bi Z.H., Huo L., Kong Q.Q., Li F., Chen J.P., Ahmad A., Wei X.X., Xie L.J., Chen C.M. (2019). Structural evolution of phosphorus species on graphene with a stabilized electrochemical interface. ACS Appl. Mater. Inter..

[63] Shumba M., Nyokong T. (2016). Development of nanocomposites of phosphorus-nitrogen co-doped graphene oxide nanosheets and nanosized cobalt phthalocyanines for electrocatalysis. Electrochim. Acta.

[64] Wen Y.Y., Rufford T.E., Hulicova-Jurcakova D., Wang L.Z. (2016). Nitrogen and phosphorous co-doped graphene monolith for supercapacitors. Chemsuschem.

[65] Yu X., Kang Y., Park H.S. (2016). Sulfur and phosphorus co-doping of hierarchically porous graphene aerogels for enhancing supercapacitor performance. Carbon.

[66] Qu K.G., Zheng Y., Zhang X.X., Davey K., Dai S., Qiao S.Z. (2017). Promotion of electrocatalytic hydrogen evolution reaction on nitrogen-doped carbon nanosheets with secondary heteroatoms. ACS Nano.

[67] Wang C.N., Luo S.Y., Yang Y.Y., Ren D.S., Yu X. (2020). Defect-rich graphene architecture induced by nitrogen and phosphorus dual doping for high-performance supercapacitors. Energy Technol.-Ger..

[68] Wang K., Li Z. (2020). Synthesis of nitrogen and phosphorus dual-doped graphene oxide as high-performance anode material for lithium-ion batteries. J. Nanosci. Nanotechol..

[69] Thirumal V., Pandurangan A., Jayavel R., Venkatesh K.S., Palani N.S., Ragavan R., Ilangovan R. (2015). Single pot electrochemical synthesis of functionalized and phosphorus doped graphene nanosheets for supercapacitor applications. J. Mater. Sci. Mater. Electron..

[70] Momodu D., Madito M.J., Singh A., Sharif F., Karan K., Trifkovic M., Bryant S., Roberts E.P.L. (2021). Mixed-acid intercalation for synthesis of a high conductivity electrochemically exfoliated graphene. Carbon.

[71] Song J., Yu Z., Gordin M.L., Hu S., Yi R., Tang D., Walter T., Regula M., Choi D., Li X. (2014). Chemically bonded phosphorus/graphene hybrid as a high performance anode for sodium-ion batteries. Nano Lett..

[72] Gong K., Du F., Xia Z., Durstock M., Dai L. (2009). Nitrogen-doped carbon nanotube arrays with high electrocatalytic activity for oxygen reduction. Science.

[73] Jiao Y., Zheng Y., Jaroniec M., Qiao S.Z. (2015). Design of electrocatalysts for oxygen- and hydrogen-involving energy conversion reactions. Chem. Soc. Rev..

[74] Zhao D., Zhuang Z., Cao X., Zhang C., Peng Q., Chen C., Li Y. (2020). Atomic site electrocatalysts for water splitting, oxygen reduction and selective oxidation. Chem. Soc. Rev..

[75] Zhang X.L., Lu Z.S., Fu Z.M., Tang Y.A., Ma D.W., Yang Z.X. (2015). The mechanisms of oxygen reduction reaction on phosphorus doped graphene: A first-principles study. J Power Sources.

[76] Bai X.W., Zhao E.J., Li K., Wang Y., Jiao M.G., He F., Sun X.X., Sun H., Wu Z.J. (2016). Theoretical insights on the reaction pathways for oxygen reduction reaction on phosphorus doped graphene. Carbon.

[77] Lei W., Deng Y.P., Li G.R., Cano Z.P., Wang X.L., Luo D., Liu Y.S., Wang D.L., Chen Z.W. (2018). Two-dimensional phosphorus-doped carbon nanosheets with tunable porosity for oxygen reactions in zinc-air batteries. ACS Catal..

[78] Balaji S.S., Ganesh P.A., Moorthy M., Sathish M. (2020). Efficient electrocatalytic activity for oxygen reduction reaction by phosphorus-doped graphene using supercritical fluid processing. B Mater. Sci..

[79] Poon K.C., Wan W.Y., Su H.B., Sato H. (2021). One-minute synthesis via electroless reduction of amorphous phosphorus-doped graphene for oxygen reduction reaction. ACS Appl. Energy Mater..

[80] Yang N., Zheng X., Li L., Li J., Wei Z. (2017). Influence of phosphorus configuration on electronic structure and oxygen reduction reactions of phosphorus-doped graphene. J. Phys. Chem. C.

[81] Dong L., Hu C., Huang X., Chen N., Qu L. (2015). One-pot synthesis of nitrogen and phosphorus co-doped graphene and its use as high-performance electrocatalyst for oxygen reduction reaction. Chem.—Asian J..

[82] Li R., Wei Z., Gou X. (2015). Nitrogen and phosphorus dual-doped graphene/carbon nanosheets as bifunctional electrocatalysts for oxygen reduction and evolution. ACS Catal..

[83] Jang D., Lee S., Kim S., Choi K., Park S., Oh J., Park S. (2018). Production of P, N co-doped graphene-based materials by a solution process and their electrocatalytic performance for oxygen reduction reaction. ChemNanoMat.

[84] Zhou L.J., Zhang C.Y., Cai X.Y., Qian Y., Jiang H.F., Li B.S., Lai L.F., Shen Z.X., Huang W. (2018). N, P co-doped hierarchical porous graphene as a metal-free bifunctional air cathode for Zn-air batteries. Chemelectrochem.

[85] Ge L.P., Wang D., Yang P.X., Xu H., Xiao L.H., Zhang G.X., Lu X.Y., Duan Z.Z., Meng F., Zhang J.Q. (2019). Graphite N-C-P dominated three-dimensional nitrogen and phosphorus co-doped holey graphene foams as high-efficiency electrocatalysts for Zn-air batteries. Nanoscale.

[86] Zhang X.R., Zhang X., Xiang X., Pan C., Meng Q.H., Hao C., Tian Z.Q., Shen P.K., Jiang S.P. (2021). Nitrogen and phosphate co-doped graphene as efficient bifunctional electrocatalysts by precursor modulation strategy for oxygen reduction and evolution reactions. Chemelectrochem.

[87] Qiao X.C., Liao S.J., You C.H., Chen R. (2015). Phosphorus and nitrogen dual doped and simultaneously reduced graphene oxide with high surface area as efficient metal-free electrocatalyst for oxygen reduction. Catalysts.

[88] Chai G.L., Qiu K.P., Qiao M., Titirici M.M., Shang C.X., Guo Z.X. (2017). Active sites engineering leads to exceptional ORR and OER bifunctionality in P, N co-doped graphene frameworks. Energy Environ. Sci..

[89] Yang Z.R., Wu J., Zheng X.J., Wang Z.J., Yang R.Z. (2015). Enhanced catalytic activity for the oxygen reduction reaction with co-doping of phosphorus and iron in carbon. J. Power Sources.

[90] Razmjooei F., Singh K.P., Bae E.J., Yu J.S. (2015). A new class of electroactive Fe- and P-functionalized graphene for oxygen reduction. J. Mater. Chem. A.

[91] Qiao X.C., Peng H.L., You C.H., Liu F.F., Zheng R.P., Xu D.W., Li X.H., Liao S.J. (2015). Nitrogen, phosphorus and iron doped carbon nanospheres with high surface area and hierarchical porous structure for oxygen reduction. J. Power Sources.

[92] Zhang J.T., Dai L.M. (2016). Nitrogen, phosphorus, and fluorine tri-doped graphene as a multifunctional catalyst for self-powered electrochemical water splitting. Angew. Chem. Int. Ed..

[93] Dou S., Shen A.L., Ma Z.L., Wu J.H., Tao L., Wang S.Y. (2015). N-, P- and S-tridoped graphene as metal-free electrocatalyst for oxygen reduction reaction. J. Electroanal. Chem..

[94] Wang Y.S., Zhang B.W., Xu M.H., He X.Q. (2015). Tunable ternary (P, S, N)-doped graphene as an efficient electrocatalyst for oxygen reduction reaction in an alkaline medium. RSC Adv..

[95] Chen W., Sin M., Wei P.J., Zhang Q.L., Liu J.G. (2016). Synergistic enhancement of electrocatalytic activity toward oxygen reduction reaction in alkaline electrolytes with pentabasic (Fe, B, N, S, P)-doped reduced graphene oxide. Chin. J. Chem..

[96] Lin H.L., Chu L., Wang X.J., Yao Z.Q., Liu F., Ai Y.N., Zhuang X.D., Han S. (2016). Boron, nitrogen, and phosphorous ternary doped graphene aerogel with hierarchically porous structures as highly efficient electrocatalysts for oxygen reduction reaction. New J. Chem..

[97] Dong F., Cai Y.X., Liu C., Liu J.Y., Qiao J.L. (2018). Heteroatom (B, N and P) doped porous graphene foams for efficient oxygen reduction reaction electrocatalysis. Int. J. Hydrog. Energy.

[98] Liu J., Zhu Y.Y., Du F.L., Jiang L.H. (2019). Iron/nitrogen/phosphorus co-doped three-dimensional porous carbon as a highly efficient electrocatalyst for oxygen reduction reaction. J. Electrochem. Soc..

[99] Yang R., Xie J.F., Liu Q., Huang Y.Y., Lv J.Q., Ghausi M.A., Wang X.Y., Peng Z., Wu M.X., Wang Y.B. (2019). A trifunctional ni-n/p-o-codoped graphene electrocatalyst enables dual-model rechargeable Zn-CO2/ Zn-O2batteries. J. Mater. Chem. A.

[100] Siahkalroudi Z.M., Aghabarari B., Vaezi M., Rodriguez-Castellon E., Martinez-Huerta M.V. (2021). Effect of secondary heteroatom (S, P) in N-doped reduced graphene oxide catalysts to oxygen reduction reaction. Mol. Catal..

[101] Yao S.X., Lyu D.D., Wei M., Chu B.X., Huang Y.L., Pan C., Zhang X.R., Tian Z.Q., Shen P.K. (2021). N, S, P co-doped graphene-like carbon nanosheets developed via in situ engineering strategy of carbon p(z)-orbitals for highly efficient oxygen redox reaction. Flatchem.

[102] Nguyen D.C., Tran D.T., Doan T.L.L., Kim N.H., Lee J.H. (2019). Constructing MoPx@MnPy heteronanoparticle-supported mesoporous N, P-codoped graphene for boosting oxygen reduction and oxygen evolution reaction. Chem. Mater..

[103] Guo W.H., Ma X.X., Zhang X.L., Zhang Y.Q., Yu D.L., He X.Q. (2016). Spinel CoMn_2_O_4_ nanoparticles supported on a nitrogen and phosphorus dual doped graphene aerogel as efficient electrocatalysts for the oxygen reduction reaction. RSC Adv..

[104] Hu X.L., Dai X.H., He X.Q. (2017). A N,P-co-doped 3D graphene/cobalt-embedded electrocatalyst for the oxygen reduction reaction. New J. Chem..

[105] Jiang H., Li C., Shen H.B., Liu Y.S., Li W.Z., Li J.E. (2017). Supramolecular gel-assisted synthesis Co2P particles anchored in multielement co-doped graphene as efficient bifunctional electrocatalysts for oxygen reduction and evolution. Electrochim. Acta.

[106] Xuan L.L., Liu X.J., Wang X. (2019). Cobalt phosphate nanoparticles embedded nitrogen and phosphorus-codoped graphene aerogels as effective electrocatalysts for oxygen reduction. Front. Mater..

[107] Wang R., Dong X.Y., Du J., Zhao J.Y., Zang S.Q. (2018). MOF-derived bifunctional Cu3P nanoparticles coated by a N, P-codoped carbon shell for hydrogen evolution and oxygen reduction. Adv. Mater..

[108] Wu D.Y., Zhu C., Shi Y.T., Jing H.Y., Hu J.W., Song X.D., Si D.H., Liang S.X., Hao C. (2019). Biomass-derived multilayer-graphene-encapsulated cobalt nanoparticles as efficient electrocatalyst for versatile renewable energy applications. ACS Sustain. Chem. Eng..

[109] Hao X.Q., Jiang Z.Q., Zhang B.A., Tian X.N., Song C.S., Wang L.K., Maiyalagan T., Hao X.G., Jiang Z.J. (2021). N-doped carbon nanotubes derived from graphene oxide with embedment of FeCo nanoparticles as bifunctional air electrode for rechargeable liquid and flexible all-solid-state zinc-air batteries. Adv. Sci..

[110] Ni B.X., Chen R., Wu L.M., Sun P.C., Chen T.H. (2021). Encapsulated FeP nanoparticles with in-situ formed P-doped graphene layers: Boosting activity in oxygen reduction reaction. Sci. China-Mater..

[111] Choi C.H., Chung M.W., Kwon H.C., Park S.H., Woo S.I. (2013). B, N- and P, N-doped graphene as highly active catalysts for oxygen reduction reactions in acidic media. J. Mater. Chem. A.

[112] Gracia-Espino E. (2016). Behind the synergistic effect observed on phosphorus nitrogen codoped graphene during the oxygen reduction reaction. J. Phys. Chem. C.

[113] Han C.L., Chen Z.Q. (2020). The mechanism study of oxygen reduction reaction (ORR) on non-equivalent P, N co-doped graphene. Appl. Surf. Sci..

[114] Zheng X.J., Yang Z.R., Wu J., Jin C., Tian J.H., Yang R.Z. (2016). Phosphorus and cobalt co-doped reduced graphene oxide bifunctional electrocatalyst for oxygen reduction and evolution reactions. RSC Adv..

[115] Hellgren N., Berlind T., Gueorguiev G.K., Johansson M.P., Stafström S., Hultman L. (2004). Fullerene-like bcn thin films: A computational and experimental study. Mater. Sci. Eng. B.

[116] Broitman E., Gueorguiev G.K., Furlan A., Son N.T., Gellman A.J., Stafström S., Hultman L. (2008). Water adsorption on fullerene-like carbon nitride overcoats. Thin Solid Film..

[117] Gueorguiev G.K., Goyenola C., Schmidt S., Hultman L. (2011). Cfx: A first-principles study of structural patterns arising during synthetic growth. Chem. Phys. Lett..

[118] Nan H.Y., Ni Z.H., Wang J., Zafar Z., Shi Z.X., Wang Y.Y. (2013). The thermal stability of graphene in air investigated by raman spectroscopy. J. Raman Spectrosc..

